# The Effect of Parental Psychological Control on Real-Life Social Avoidance in College Students: A Moderated Chain Mediation Model

**DOI:** 10.3390/bs16010034

**Published:** 2025-12-23

**Authors:** Panqin Ye, Lin Zhang, Yan Li, Furong Lu, Yufang Bian

**Affiliations:** 1 School of Educational Science, Shanxi University, Taiyuan 030006, China; yepanqinzi@163.com (P.Y.); zhanglin7@sxu.edu.cn (L.Z.); lufr@sxu.edu.cn (F.L.); 2 Faculty of Education, The Chinese University of Hong Kong, Hong Kong 999077, China; li-yan@link.cuhk.edu.hk; 3 Collaborative Innovation Center of Assessment Toward Basic Education Quality, Beijing Normal University, Beijing 100875, China

**Keywords:** parental psychological control, social avoidance, basic psychological needs, problematic smartphone use, interpersonal sensitivity

## Abstract

This study examined the associations between parental psychological control and real-life social avoidance among college students, focusing on the chain mediating roles of basic psychological needs and problematic smartphone use, as well as the moderating role of interpersonal sensitivity. A total of 1879 college students participated in the study. The results revealed that parental psychological control is positively associated with college students’ social avoidance behavior. Furthermore, basic psychological needs and problematic smartphone use played significant sequential mediating roles in this association. Moreover, a sequential mediation pathway was found through basic psychological needs and problematic smartphone use. Additionally, interpersonal sensitivity was identified as a significant moderator, moderating the strength of the association between parental psychological control, and basic psychological needs. These findings provide important evidence for understanding the correlational mechanisms associated with real-life social avoidance among college students and offer practical insights for the development of intervention strategies.

## 1. Introduction

During college life, social interaction plays a vital role in shaping individuals’ social functioning and healthy development. In China, the rapid growth of the internet and individuals’ strong engagement with online environments have been linked to increased tendencies toward real-life social avoidance (Tan et al., 2025). According to a report by China Youth Daily, more than 80% of Chinese college students reported experiencing mild “social anxiety” (Cheng et al., 2021), exhibiting heightened withdrawal tendencies in social settings and reduced participation in everyday interactions. Social avoidance refers to negative behavioral tendencies in interpersonal contexts, typically manifested as avoiding contact or conversation with others, withdrawing from social situations, and experiencing negative affect during interactions (Watson & Friend, 1969). Individuals with higher levels of social avoidance face greater risks in interpersonal adaptation, which may lead to a range of social adjustment difficulties (Zhu et al., 2022). Prior studies have shown that socially avoidant individuals report higher levels of loneliness (Lemieux et al., 2013) and lower levels of self-esteem (Shang et al., 2025). Social avoidance has also been associated with elevated risks of depression, anxiety, and other mental health problems (Tu et al., 2025; Yuan et al., 2022). As the frequency of social interactions decreases, social competence declines accordingly, reinforcing avoidance behaviors and creating a vicious cycle. Given these risks, it is essential to examine the factors contributing to college students’ real-life social avoidance.

### 1.1. The Relationship Between Parental Psychological Control and Social Avoidance

Grounded in Bronfenbrenner’s (1979) ecological systems theory, the family—as the primary microsystem—is a crucial determinant of developmental outcomes. Within this context, parents exert a lasting association with their children’s social competencies and adaptive behaviors. Among parenting practices, parental psychological control—an intrusive form of parenting—has been consistently linked to social maladjustment (Zhou et al., 2024). Meta-analytic findings have indicated that parental psychological control exhibits a moderate effect size in relation to problem behaviors (r = 0.218; Yan et al., 2020) and internalizing symptoms (r = 0.27; Salaam & Kyere, 2025), highlighting its potential role in the development of avoidant behaviors. Psychological control is typically manifested through tactics such as guilt induction, love withdrawal, and authority assertion, through which parents regulate their children’s thoughts, emotions, and behaviors (Q. Wang et al., 2007). In doing so, parents often substitute emotional manipulation for respect toward their children’s individuality.

Parents with high levels of psychological control are generally less sensitive and responsive to their children’s needs, which is related to the development of insecure attachment relationships (Barber, 1996). Early attachment patterns play a crucial role in shaping later interpersonal relationships (De Paoli et al., 2017). Insecure attachment, for example, may hinder the development of interpersonal trust during subsequent socialization, often leading to self-doubt in social interactions. Moreover, controlling parenting undermines children’s autonomy (Soenens et al., 2005). High levels of parental psychological control can obstruct psychological autonomy (Chen et al., 2021), foster negative self-schemas, and increase vulnerability to internalizing problems such as anxiety and depression (Chyung et al., 2022), thereby heightening social anxiety (Zhou et al., 2024). Social avoidance, in turn, represents a typical behavioral manifestation of social anxiety. China is a society characterized by collectivist cultural values, in which parents are traditionally ascribed the authority to supervise and guide their children. This cultural context places strong emphasis on filial piety and the value of obedience (Chao, 1994; Triandis, 1995). Cross-cultural research has consistently demonstrated that Chinese parents tend to exert higher levels of psychological control (Ng et al., 2014; Q. Wang et al., 2007). Notably, existing studies on parental psychological control and social avoidance have largely focused on adolescents, while investigations targeting college students remain relatively scarce. Given the unique developmental tasks of this stage, the present study examines the effects of parental psychological control on real-life social avoidance among college students and its underlying mechanism.

### 1.2. The Mediating Role of Basic Psychological Needs

Grounded in self-determination theory (Deci & Ryan, 2012; Ryan et al., 2022), the satisfaction of basic psychological needs—autonomy, competence, and relatedness—is directly associated with individuals’ mental health and social adaptation. When these needs are chronically unmet, psychological development becomes hindered. Previous research has shown that parental psychological control directly threatens the fulfillment of these needs, resulting in need thwarting (C. Peng et al., 2024). Within highly controlling family environments, college students often exhibit suppressed emotional expression and a diminished sense of agency, leading to concurrent undermining of autonomy and competence (Chen et al., 2021). According to self-determination theory (Deci & Ryan, 2012), such parenting styles prevent individuals from satisfying their needs for autonomy, competence, and relatedness, thereby producing deficits in cognitive functioning, interpersonal relationships, and emotional regulation (Soenens & Vansteenkiste, 2010). When basic psychological needs are not satisfied, individuals experience continuous declines in self-esteem (Butkovic et al., 2020; Yang et al., 2024) and develop negative self-perceptions in social interactions, which substantially increase the likelihood of social avoidance behaviors. The undermining of autonomy and competence reduces individuals’ confidence in their abilities, discouraging self-expression in social contexts and prompting withdrawal from interpersonal interactions. This withdrawal, in turn, further undermines relatedness satisfaction, creating a cycle of persistent need thwarting that impedes healthy social functioning. Thus, parental psychological control may indirectly influence college students’ real-life social avoidance through the undermining of basic psychological needs.

### 1.3. The Mediating Role of Problematic Smartphone Use

The negative effects of parental psychological control are long-lasting, with early perceptions of such control during adolescence potentially leading to maladjustment in adulthood (Loeb et al., 2021). A recent systematic review indicates that problematic smartphone use has a high prevalence among this population and is associated with various negative psychological and adjustment outcomes, providing an important background for exploring its underlying mechanisms in this study (Sanchez-Fernandez & Borda-Mas, 2023). Problematic smartphone use, as one form of maladaptive coping, is defined as uncontrolled and excessive use of mobile phones that produces harmful consequences for both physical and psychological health (Billieux, 2012; X. Liu et al., 2022b). Some scholars also conceptualize this behavior as smartphone addiction or smartphone dependence (J. H. Kim, 2018). Prior research has confirmed a positive association between controlling parenting and adolescents’ smartphone addiction (Lian et al., 2016). By constraining the development of self-regulation (X. Li et al., 2013), parental psychological control makes it more difficult for individuals to resist temptations. Under high parental control, heightened psychological stress may drive individuals to use smartphones as a means of temporarily escaping real-life pressures (Van Ingen et al., 2015). However, prolonged exposure to such parenting environments poses severe threats to psychological health (X. Liu et al., 2022a). Findings from Abu Khait et al. (2024) further indicated that smartphone addiction may significantly exacerbate the development of social anxiety. For individuals with high levels of problematic smartphone use, time spent on mobile devices largely replaces face-to-face interactions, hindering the development of real-life social competence—an ability that is particularly crucial in college life. At the same time, parental psychological control weakens social skills, making it difficult for individuals to integrate with peers (J. Kim & Cicchetti, 2010). Under these influences, college students are more likely to exhibit withdrawal and avoidance in interpersonal contexts. Thus, parental psychological control may indirectly influence college students’ real-life social avoidance through problematic smartphone use.

### 1.4. The Chain Mediating Role of Basic Psychological Needs and Problematic Smartphone Use

The compensatory internet use theory (Kardefelt-Winther, 2014) posits that individuals may seek to compensate in online environments for needs that are unmet in real life. When basic psychological needs are not satisfied in everyday contexts, individuals tend to pursue alternative avenues of fulfillment. Smartphones, now the primary gateway to the internet, offer both convenience and diverse content, making them a preferred medium for compensation (Y. Liu et al., 2016). Once individuals experience gratification through smartphone use, the tendency to seek need fulfillment via digital means becomes reinforced (Wong et al., 2015), leading to increased smartphone use and ultimately greater dependence. Previous studies have shown that individuals experiencing thwarting of basic psychological needs are more likely to display internet-related problematic behaviors (Celikkaleli & Ata, 2025; Kurker & Surucu, 2024). Accordingly, parental psychological control may indirectly influence college students’ real-life social avoidance through a sequential mediating pathway involving basic psychological needs and problematic smartphone use.

### 1.5. The Moderating Role of Interpersonal Sensitivity

Although the association between parental psychological control and basic psychological needs has been widely discussed (Barber, 1996; Soenens & Vansteenkiste, 2010), relatively limited attention has been paid to the role of individual differences in this association. Interpersonal sensitivity, as an important dispositional characteristic, may be relevant in this context. Some scholars conceptualize interpersonal sensitivity as a positive social–perceptual capacity involving the accurate perception and evaluation of others’ thoughts, emotions, and traits (Hall et al., 2009; Snodgrass, 1985). Individuals with higher interpersonal sensitivity often display greater emotional resonance with others (Kou & Wang, 2023; Bernieri, 2001). At the same time, a substantial body of research tends to describe interpersonal sensitivity as a stable personality characteristic characterized by heightened vigilance toward negative social evaluation and pronounced sensitivity to others’ behaviors and emotions (Boyce & Parker, 1989). Individuals high in this trait are more likely to display negative attributional tendencies (Marian, 2013), which are often accompanied by stronger emotional distress in autonomy-related contexts. In the present study, interpersonal sensitivity is defined in accordance with Boyce and Parker (1989) as individuals’ heightened vulnerability to rejection, disapproval, and relational threats in social contexts. From the perspective of the stress–diathesis model (Monroe & Simons, 1991), interpersonal sensitivity can be conceptualized as a relatively stable individual vulnerability, whereas parental psychological control constitutes a chronic source of family-related psychological stress. The interaction between these two factors is jointly associated with individuals’ levels of basic psychological need satisfaction and, in turn, exerts profound effects on their behavioral adaptation. Therefore, it is necessary to examine the moderating role of interpersonal sensitivity while investigating the mechanisms through which parental psychological control affects individuals’ behavioral outcomes.

### 1.6. The Present Study

**Figure 1:**
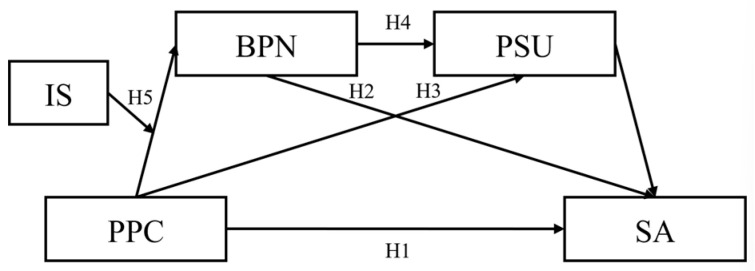
Hypothetical Model. Note: PPC, Parental Psychological Control; SA, Social Avoidance; BPN, Basic Psychological Needs; PSU, Problematic Smartphone Use; IS, Interpersonal Sensitivity.

The present study constructed a moderated chain mediation model (Figure 1) to examine the relationships among parental psychological control, basic psychological needs, problematic smartphone use, and real-life social avoidance among college students. Correlational analyses were first conducted to preliminarily test the associations among variables. Mediation analyses were then employed to examine the mediating roles of basic psychological needs and problematic smartphone use in the relationship between parental psychological control and real-life social avoidance. Finally, moderated chain mediation analyses were performed to test whether interpersonal sensitivity moderated the effect of parental psychological control on basic psychological needs.

Based on the above theoretical and empirical considerations, the following hypotheses were proposed. Hypothesis 1: Parental psychological control is positively associated with real-life social avoidance in college students. Hypothesis 2: Basic psychological needs mediate the relationship between parental psychological control and real-life social avoidance. Hypothesis 3: Problematic smartphone use mediates the relationship between parental psychological control and real-life social avoidance. Hypothesis 4: Basic psychological needs and problematic smartphone use sequentially mediate the relationship between parental psychological control and real-life social avoidance. Hypothesis 5: Interpersonal sensitivity moderates the effect of parental psychological control on basic psychological needs.

## 2. Methods

### 2.1. Participants

Data were collected in 2025 using a convenience sampling approach through the online survey platform. The research team distributed the questionnaire invitation via Enterprise WeChat in official class groups, and students voluntarily completed the survey by accessing the provided link. A total of 1503 questionnaires were obtained. After excluding invalid responses based on completion time, attention-check items, and response consistency, 1413 valid questionnaires were retained (effective response rate = 75.20%). Gender was coded as 1 = male and 2 = female. The final sample included 608 males (43.03%) and 805 females (56.97%). Regarding academic year, 702 participants (49.70%) were freshmen, 335 (23.70%) were sophomores, 209 (14.80%) were juniors, and 166 (11.70%) were seniors. In terms of family structure, 1312 participants (92.90%) were from two-parent families, whereas 101 (7.10%) were from single-parent families. With respect to residential background, 654 participants (46.3%) were from urban areas and 759 (53.70%) were from rural areas. Regarding academic major, 398 participants were in humanities and social sciences (28.20%), 912 were in science and engineering (64.50%), and 102 were in other fields (7.30%). Concerning parental education, 378 participants reported that at least one parent had received higher education (26.70%), whereas 1036 reported that neither parent had received higher education (73.30%). Before completing the questionnaire, participants were informed of the purpose of the study, the anonymity of their responses, and their right to withdraw at any time. No additional material compensation was provided for participation.

### 2.2. Measures

#### 2.2.1. Parental Psychological Control Scale (PPCS)

Parental psychological control was assessed with the Parental Psychological Control Scale developed by Q. Wang et al. (2007). The scale consists of 18 items covering three dimensions: guilt induction, love withdrawal, and authority assertion. All items were rated on a 5-point Likert scale ranging from 1 (strongly disagree) to 5 (strongly agree), with higher scores indicating stronger parental psychological control. In the present study, Cronbach’s *α* coefficient was 0.94. Confirmatory factor analysis demonstrated good structural validity (*χ*^2^/*df* = 4.87, CFI = 0.96, TLI = 0.94, RMSEA = 0.05).

#### 2.2.2. Basic Psychological Needs Scale (BPNS)

Basic psychological needs were assessed using the Basic Psychological Needs Scale originally developed by Gagné (2003) and later revised by J. S. Liu et al. (2013). The scale consists of 19 items measuring three dimensions: competence, autonomy, and relatedness. Items were rated on a 7-point Likert scale (1 = “strongly disagree” to 7 = “strongly agree”), with higher scores reflecting greater satisfaction of basic psychological needs. In the present study, Cronbach’s *α* coefficient was 0.87. Confirmatory factor analysis results indicated good structural validity (*χ*^2^/*df* = 4.52, CFI = 0.93, TLI = 0.90, RMSEA = 0.05).

#### 2.2.3. Smartphone Addiction Scale (SAS)

Problematic smartphone use was assessed using the Smartphone Addiction Scale developed by Kwon et al. (2013) and revised by Xiang et al. (2019). The scale includes 32 items covering six dimensions: daily life disturbance, positive anticipation, withdrawal, cyberspace-oriented relationship, overuse, and tolerance. Responses were rated on a 6-point Likert scale (1 = “strongly disagree” to 6 = “strongly agree”), with higher scores indicating a higher tendency toward smartphone addiction. In the present study, Cronbach’s *α* coefficient was 0.94. Confirmatory factor analysis results indicated good structural validity (*χ*^2^/*df* = 4.37, CFI = 0.91, TLI = 0.90, RMSEA = 0.05).

#### 2.2.4. Social Avoidance and Distress Scale (SADS)

Real-life social avoidance was assessed using the Social Avoidance and Distress Scale developed by Watson and Friend (1969) and revised by C. Z. Peng et al. (2003). The scale consists of 28 items measuring two dimensions: social avoidance and social distress. Items were rated on a 5-point Likert scale (1 = “strongly disagree” to 5 = “strongly agree”), with higher scores indicating greater levels of social avoidance and distress. In the present study, Cronbach’s *α* coefficient was 0.94. Confirmatory factor analysis results showed good structural validity (*χ*^2^/*df* = 4.95, CFI = 0.94, TLI = 0.92, RMSEA = 0.05).

#### 2.2.5. Interpersonal Sensitivity Scale (ISS)

Interpersonal sensitivity was assessed using the Interpersonal Sensitivity Scale developed by Boyce and Parker (1989) and revised by Q. Li and Wang (2019). The scale consists of 19 items across five dimensions: fragile inner self, separation anxiety, interpersonal awareness, need for approval, and friendly consideration. Items were rated on a 4-point Likert scale (1 = “strongly disagree” to 4 = “strongly agree”), with higher scores indicating higher interpersonal sensitivity. In the present study, Cronbach’s *α* coefficient was 0.90. Confirmatory factor analysis results indicated good structural validity (*χ*^2^/*df* = 4.86, CFI = 0.93, TLI = 0.92, RMSEA = 0.05).

### 2.3. Data Analysis

Statistical analyses were conducted using SPSS 25.0 and Mplus 8. First, tests for common method bias, descriptive statistics, and correlation analyses were performed, and confirmatory factor analyses were conducted for each scale. Second, regression analyses were used to examine the relationships among parental psychological control, basic psychological needs, problematic smartphone use, and social avoidance in order to test the proposed hypotheses. Finally, by incorporating interaction terms into the statistical model, we further examined the moderating role of interpersonal sensitivity in the relationship between parental psychological control and basic psychological needs. Post hoc power analyses for the moderated mediation model were conducted using the *semPower* package in R 4.3.3., based on a sample size of 1413 and the standardized path coefficients.

## 3. Results

### 3.1. Common Method Bias Test

Since all measures in the present study were self-report questionnaires, there was a potential risk of common method bias. Therefore, Harman’s single-factor test was conducted to examine common method variance. The results showed that a total of 21 factors had eigenvalues greater than 1. The first factor accounted for 19.99% of the variance, which was below the critical threshold of 40%. These results suggest that common method bias was not a major concern in the present study.

### 3.2. Descriptive Statistics and Correlation Analysis

The results of the correlation analysis (Table 1) showed that parental psychological control was positively correlated with social avoidance, problematic smartphone use, and interpersonal sensitivity (*p* < 0.001). Social avoidance was also positively correlated with problematic smartphone use and interpersonal sensitivity (*p* < 0.001), and a significant positive correlation was observed between problematic smartphone use and interpersonal sensitivity (*p* < 0.001). In contrast, basic psychological needs were negatively correlated with parental psychological control, social avoidance, problematic smartphone use, and interpersonal sensitivity (*p* < 0.001). In addition, both gender and age were positively correlated with problematic smartphone use (*p* < 0.05).

### 3.3. Mediation Effect Analysis

Model 6 of PROCESS 4.1 in SPSS 25.0 was used to test the chain mediation model, controlling for age and gender. As shown in Table 2, when basic psychological needs and problematic smartphone use were included as mediating variables, the direct relationship between parental psychological control and social avoidance was not significant (β = 0.013, *p* > 0.05). Parental psychological control was negatively associated with basic psychological needs (β = −0.300, *p* < 0.001) and positively was positively associated with problematic smartphone use (β = 0.246, *p* < 0.001). Basic psychological needs were negatively associated with problematic smartphone use (β = −0.272, *p* < 0.001) and social avoidance (β = −0.486, *p* < 0.001). Problematic smartphone use was positively associated with social avoidance (β = 0.066, *p* < 0.01).

The bias-corrected percentile bootstrap method further revealed that the mediating effect of basic psychological needs on the relationship between parental psychological control and social avoidance was significant, with an effect size of 0.146, accounting for 80.663% of the total effect. The mediating effect of problematic smartphone use was also significant, with an effect size of 0.016, accounting for 8.840% of the total effect. In addition, the chain mediation effect of basic psychological needs and problematic smartphone use on the relationship between parental psychological control and social avoidance was significant, with an effect size of 0.005, accounting for 2.762% of the total effect (Table 3).

### 3.4. Moderation Effect Analysis

Model 83 of PROCESS 4.1 in SPSS 25.0 was employed to test the moderated chain mediation model, controlling for gender and grade. The results (Table 4) showed that the interaction term of parental psychological control and interpersonal sensitivity was significantly associated with basic psychological needs (β = 0.126, *p* < 0.01), indicating that interpersonal sensitivity moderated the effect of parental psychological control on basic psychological needs.

**Figure 2:**
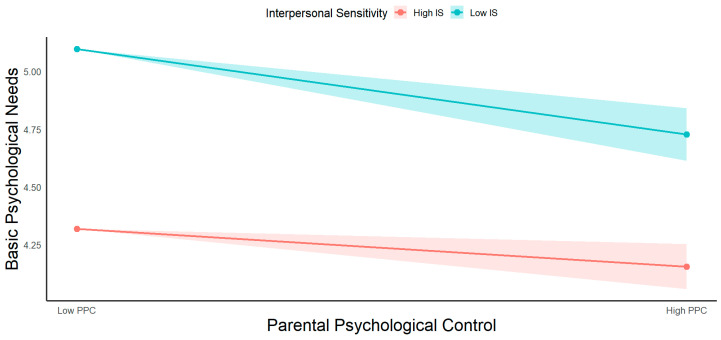
Moderating effect of Interpersonal Sensitivity. Note: PPC, Parental Psychological Control; BPN, Basic Psychological Needs; IS, Interpersonal Sensitivity.

Furthermore, to further illustrate the interactive effect of parental psychological control and interpersonal sensitivity on basic psychological needs, a simple slope analysis was conducted (Figure 2). Although parental psychological control was negatively associated with basic psychological needs satisfaction at both high and low levels of interpersonal sensitivity, these associations were weaker at high levels (β = −0.095, t = −3.281, *p* < 0.01) compared to low levels (β = −0.214, t = −6.351, *p* < 0.001). These findings suggest that higher interpersonal sensitivity attenuated the negative association between parental psychological control and basic psychological needs. Post hoc power analysis revealed that the statistical power for all key paths were higher than 0.8, indicating that the sample size was sufficient to detect the effects with high precision.

## 4. Discussion

This study employed a moderated chain mediation model to investigate the influence of parental psychological control on college students’ real-life social avoidance. The analysis focused on the mediating roles of basic psychological needs and problematic smartphone use and the moderating role of interpersonal sensitivity, offering deeper insight into the mechanisms linking parental psychological control to social avoidance.

### 4.1. The Effect of Parental Psychological Control on Social Avoidance

The present study found a significant positive association between parental psychological control and real-life social avoidance, supporting Hypothesis 1. This result indicates that parental psychological control is significantly associated with college students’ avoidance of social interactions in real-life contexts. Consistent with previous research, the finding further confirms the detrimental role of parental psychological control in individual social adaptation (Barber, 1996). Through strategies such as guilt induction and love withdrawal, parental psychological control undermines autonomy (Soenens et al., 2005), leaving individuals feeling helpless and insecure in social situations. Such psychological pressure may prompt students to deliberately avoid social interactions in order to minimize potential negative evaluations or emotional harm. In addition, parental psychological control may indirectly exacerbate social avoidance by compromising the security of parent–child relationships. According to attachment theory (Bowlby, 1982; Zhang et al., 2022), controlling parenting behaviors may foster insecure attachment patterns, which in turn extend to other social relationships, manifesting as generalized distrust and avoidance in interpersonal interactions (Gazelle & Rubin, 2019). Importantly, the effects of parental psychological control on social avoidance may be both long-lasting and cumulative. Prior studies have shown that psychologically controlling parenting fosters tendencies toward self-deprecation (Man et al., 2015), which increases the likelihood of developing negative social expectations and, consequently, enduring tendencies toward real-life social avoidance even in adulthood.

### 4.2. The Mediating Roles of Basic Psychological Needs and Problematic Smartphone Use

The association between parental psychological control and real-life social avoidance is not merely a direct effect but rather unfolds through a series of complex psychological and behavioral mechanisms. The present study revealed that thwarting of basic psychological needs and problematic smartphone use together form a complete chain mediation pathway.

Specifically, the findings indicated that basic psychological needs mediated the relationship between parental psychological control and real-life social avoidance, supporting Hypothesis 2. According to the theory of basic psychological needs, individuals possess an innate tendency toward self-development, and the needs for competence, autonomy, and relatedness constitute the core driving forces of psychological functioning (Deci & Ryan, 2012). Parental psychological control, through strategies such as guilt induction, love withdrawal, and authoritarianism, undermines the satisfaction of these needs. Restricting children’s freedom of expression threatens their autonomy; critical or demeaning parenting styles hinder competence satisfaction (Costa et al., 2015), and controlling practices foster insecure attachment patterns that undermine the need for relatedness (Assor et al., 2004). Such need thwarting not only triggers immediate emotional distress—manifested as anxiety and self-doubt—but may also lead to long-term depletion of psychological resources. These findings further support self-determination theory, which posits that chronic need thwarting results in maladaptive functioning (Vansteenkiste & Ryan, 2013). When basic psychological needs are thwarted, individuals may experience diminished self-efficacy. Loss of competence fosters negative expectations of social contexts, prompting avoidance of interactions that could expose perceived inadequacies. At the same time, thwarting of relatedness needs generalizes relationship anxiety, fostering distrust in interpersonal connections and projecting parental control patterns onto broader social relationships (La Guardia et al., 2000).

The present study found that parental psychological control was indirectly associated with college students’ real-life social avoidance through the mediating role of problematic smartphone use, supporting Hypothesis 3. The positive predictive effect of parental psychological control on problematic smartphone use is consistent with prior research (J. Wang et al., 2025). Problematic smartphone use or smartphone addiction is typically associated with elevated psychological stress and negative emotions. When faced with high levels of parental control, college students may turn to smartphones as an immediate tool for emotion regulation. By immersing themselves in social media, online games, or short videos, individuals can temporarily escape real-life problems and pressures, thereby obtaining short-term psychological relief (Zhang et al., 2024). However, while such avoidance behaviors may temporarily alleviate negative emotions, they often lead to long-term psychological dependence. Kaibin et al. (2025) suggested that smartphone dependency is associated with neglect of real-life social interactions, thereby generating interpersonal difficulties. Algorithm-driven online interactions can briefly activate the brain’s reward system (Meshi et al., 2015), yet this immediate gratification creates operant conditioning effects, making individuals increasingly reliant on smartphones to cope with stress rather than developing real-life social skills. Over time, the low-risk nature of online interaction, contrasted with the uncertainty inherent in face-to-face social contexts, further exacerbates tendencies toward real-life social avoidance. Moreover, individuals with high levels of smartphone dependency are more likely to experience social anxiety (Wei et al., 2024), which, in turn, intensifies their avoidance of real-life social situations.

The study further confirmed the sequential mediating roles of basic psychological needs and problematic smartphone use in the relationship between parental psychological control and real-life social avoidance, supporting Hypothesis 4. Specifically, individuals experiencing high levels of parental psychological control suffer from unmet basic psychological needs, which in turn foster smartphone addiction and ultimately contribute to real-life social avoidance. This finding offers a novel theoretical framework for understanding the mechanisms underlying college students’ real-life social avoidance and provides empirical support for the compensatory internet use theory proposed by Kardefelt-Winther (2014). Parental psychological control directly threatens students’ autonomy, competence, and relatedness, leading to need thwarting. When such needs are unmet within the family context, students may turn to smartphones as a compensatory mechanism. For example, the ability to choose content and present oneself online may serve as a substitute for autonomy, while receiving likes or positive feedback can enhance a sense of competence (Parent, 2023). The online environment alleviates the distress and pressure associated with unmet needs in real life (Kardefelt-Winther, 2014). Over time, however, the reliance on smartphones to satisfy unmet basic psychological needs fosters digital dependency, shifting usage patterns from “instrumental use” to “addictive use”. As Yeh et al. (2008) noted, high levels of smartphone addiction reduce real-life social interactions, thereby exacerbating tendencies toward social avoidance.

Moreover, although the chain mediation effect was statistically significant, it accounted for only 2.76% of the total effect, indicating a small effect size. This suggests that the sequential pathway has limited explanatory value and should be interpreted with caution. Future studies may use longitudinal or experimental designs to further verify its robustness.

### 4.3. The Moderating Role of Interpersonal Sensitivity

The present study found that interpersonal sensitivity significantly moderated the negative the association between parental psychological control and basic psychological needs, supporting Hypothesis 5. Notably, the moderating effect was negative. Specifically, the negative predictive effect of parental psychological control on basic psychological needs was weaker among individuals with high interpersonal sensitivity, whereas the association was stronger among those with low interpersonal sensitivity. Several possible explanations may account for this finding. According to the stress–diathesis model (Monroe & Simons, 1991), interpersonal sensitivity represents a relatively stable individual vulnerability, whereas parental psychological control constitutes a persistent source of family-related stress. Individuals high in interpersonal sensitivity, due to their chronic vigilance toward rejection and negative evaluation, are more likely to develop negative self-schemas and relational expectations during early parent–child interactions (Feldman & Downey, 1994). Their level of basic psychological need satisfaction also tends to remain relatively low and shows limited potential for further decline. Thus, when parental psychological control intensifies, these individuals high in interpersonal sensitivity exhibit only slight additional reductions in need satisfaction, as their functioning is already constrained within a narrow range with little room for further deterioration. In contrast, individuals with lower interpersonal sensitivity show less defensive responding to social threat in daily contexts; when they perceive increased parental psychological control, their autonomy, competence, and relatedness are more readily undermined, resulting in a stronger negative association. Furthermore, this phenomenon may also be linked to habituation. Compared with individuals low in interpersonal sensitivity, those high in interpersonal sensitivity are more likely to develop adaptive coping mechanisms to mitigate the impact of parental psychological control over time. As a result, parental psychological control exerts a weaker influence on the basic psychological needs of individuals with high interpersonal sensitivity.

Moreover, prior research has consistently demonstrated that parental psychological control impedes the satisfaction of basic psychological needs (Soenens et al., 2015); however, individuals differ substantially in how they cognitively appraise and emotionally process parenting behaviors. The present findings further suggest that such differences are not primarily attributable to positive cognitive reappraisal or empathic tendencies but may instead stem from enduring patterns of threat processing and defensive adaptation formed across earlier developmental experiences.

Notably, interpretations of parental psychological control and social withdrawal should be situated within the Chinese cultural context. In collectivist settings, parental authority and the use of psychological control are often regarded as ordinary expressions of care and responsibility (Chao, 1994). Social withdrawal may also reflect a culturally endorsed tendency toward cautious and restrained interpersonal engagement rather than a purely maladaptive pattern. Therefore, the associations discussed in this study should be understood within this cultural framework, and caution is warranted when extending these conclusions to other cultural contexts.

### 4.4. Implications and Limitations

This study employed a moderated chain mediation model to uncover the underlying mechanisms through which parental psychological control is associated with college students’ real-life social avoidance, thereby deepening the theoretical framework linking parenting practices and adolescents’ social adaptation. These findings provide a theoretical perspective for understanding the complex interplay between family environment and individual psychological and behavioral outcomes, highlighting their theoretical significance. Moreover, the results underscore practical implications, suggesting that parents should reduce psychologically controlling practices and instead adopt autonomy-supportive and other positive parenting strategies to enhance the satisfaction of their children’s basic psychological needs, thereby reducing problematic smartphone use and tendencies toward real-life social avoidance. Meanwhile, interventions targeting college students’ social avoidance in real-life contexts should pay attention to improving the satisfaction of basic psychological needs as an essential psychological mechanism and to alleviating their smartphone addiction.

Despite its contributions, this study is subject to several limitations. First, the cross-sectional design precludes establishing causal directionality among variables. Although the theoretical model posits parental psychological control as influencing social avoidance, reverse causation remains plausible. For instance, individuals with higher social avoidance tendencies may be more sensitive to controlling cues, leading to greater reporting of parental psychological control. Future research should employ longitudinal or experimental designs to clarify causal sequencing. Second, the sample was drawn solely from universities in Shanxi Province, which may limit the generalizability of findings due to regional and cultural specificity. Within China’s collectivist cultural context, parental psychological control may be partially perceived as “care” rather than “intrusion”, differing from interpretations common in Western individualistic cultures. Thus, caution is warranted when generalizing the results to other cultural or regional contexts. Third, all measures relied on self-report questionnaires, which may be influenced by social desirability bias and subjective responses. Future research could incorporate multiple sources of data, such as peer or parent reports, behavioral observations, or experimental methods, to strengthen the reliability and validity of the findings. Finally, future studies should further investigate how parental psychological control manifests in the digital era, where technologically mediated communication may redefine the boundaries of parental influence and carry nuanced implications for college students’ social adjustment and avoidance behaviors.

## 5. Conclusions

The present study demonstrated that parental psychological control is an important predictor of college students’ real-life social avoidance, operating through a sequential mediation pathway involving basic psychological need and problematic smartphone use. Interpersonal sensitivity further emerged as a significant moderator, indicating that individual differences shape the extent to which parental psychological control affects basic psychological needs. These findings extend existing theories by integrating self-determination theory, compensatory internet use theory, and the differential susceptibility hypothesis into a unified explanatory framework, while also providing valuable guidance for family education and intervention strategies.

## Figures and Tables

**Table 1:** Descriptive statistics and correlations among main variables (*N* = 1413).

Variable	M	SD	Min	Max	Skewness	Kurtosis	1	2	3	4	5
1. PPC	2.36	0.81	1.00	5.00	0.595	−0.039	1				
2. SA	3.17	0.69	1.04	5.00	−0.114	0.103	0.212 ***	1			
3. BPN	4.59	0.81	1.16	6.89	0.193	0.288	−0.304 ***	−0.596 ***	1		
4. PSU	3.46	0.80	1.16	6.00	0.198	0.390	0.328 ***	0.282 ***	−0.345 ***	1	
5. IS	2.68	0.49	1.05	4.00	0.079	0.262	0.358 ***	0.498 ***	−0.493 ***	0.482 ***	1

Note: *** *p* < 0.001. PPC, Parental Psychological Control; SA, Social Avoidance; BPN, Basic Psychological Needs; PSU, Problematic Smartphone Use; IS, Interpersonal Sensitivity.

**Table 2:** Test for the chain mediation model.

Regression Equation		Overall Fit Indices		Significance of Regression Coefficients			
Outcome Variable	Predictors	R^2^	F	β	Boot LLCI	Boot ULCI	t
BPN	Gender	0.094	48.559 ***	0.060	−0.021	0.141	1.462
	Age			0.004	−0.034	0.042	0.193
	PPC			−0.300	−0.349	−0.250	−11.923 ***
PSU	Gender	0.186	80.153 ***	0.136	0.059	0.212	3.486 ***
	Age			0.053	0.017	0.089	2.890 **
	PPC			0.246	0.197	0.294	9.869 ***
	BPN			−0.272	−0.322	−0.223	−10.842 ***
SA	Gender	0.366	162.283 ***	0.084	0.026	0.142	2.820 **
	Age			−0.001	−0.029	0.026	−0.090
	PPC			0.013	−0.025	0.052	0.675
	BPN			−0.486	−0.526	−0.447	−24.450 ***
	PSU			0.066	0.027	0.106	3.276 **

Note: ** *p* < 0.01, *** *p* < 0.001; Boot LLCI = lower limit of the 95% bootstrap confidence interval; Boot ULCI = upper limit of the 95% bootstrap confidence interval. Note: PPC, Parental Psychological Control; SA, Social Avoidance; BPN, Basic Psychological Needs; PSU, Problematic Smartphone Use.

**Table 3:** Mediating effect analysis of the chain mediating model.

Path	Effect	Percentage of Total Effect	Lower Limit of 95% CI	Upper Limit of 95% CI
Total effect	0.181		0.137	0.224
Direct effect	0.013	7.182%	−0.025	0.052
Total indirect effect	0.168	92.818%	0.136	0.199
PPC → BPN → SA	0.146	80.663%	0.118	0.179
PPC → PSU → SA	0.016	8.840%	0.005	0.028
PPC → BPN → PSU → SA	0.005	2.762%	0.002	0.01

Note: PPC, Parental Psychological Control; SA, Social Avoidance; BPN, Basic Psychological Needs; PSU, Problematic Smartphone Use.

**Table 4:** Test for the moderated chain mediation mode.

Regression Equation		Overall Fit Indices		Significance of Regression Coefficients			
Outcome Variable	Predictors	R^2^	F	β	Boot LLCI	Boot ULCI	t
BPN	Gender	0.268	103.303 ***	0.068	−0.005	0.141	1.837
	Age			−0.007	−0.041	0.028	−0.379
	PPC			−0.155	−0.203	−0.107	−6.318 ***
	IS			−0.712	−0.794	−0.634	−17.874 ***
	PPC × IS			0.126	0.04	0.207	3.024 **
PSU	Gender	0.186	80.153 ***	0.136	0.059	0.212	3.486 **
	Age			0.053	0.017	0.089	2.890 **
	PPC			0.246	0.197	0.294	9.869 ***
	BPN			−0.272	−0.322	−0.223	−10.842 ***
SA	Gender	0.366	162.283 ***	0.084	0.026	0.142	2.820 **
	Age			−0.001	−0.029	0.026	−0.090
	PPC			0.013	−0.025	0.052	0.675
	BPN			−0.486	−0.526	−0.447	−24.450 ***
	PSU			0.066	0.027	0.106	3.276 **

Note: ** *p* < 0.01, *** *p* < 0.001. PPC, Parental Psychological Control; SA, Social Avoidance; BPN, Basic Psychological Needs; PSU, Problematic Smartphone Use; IS, Interpersonal Sensitivity.

## Data Availability

The data presented in this study are available upon request from the corresponding author on reasonable request. The data are not publicly available due to confidentiality and research ethics.
